# Egler Chiari (★1934 †2020)

**DOI:** 10.1590/0037-8682-0574-2020

**Published:** 2020-08-26

**Authors:** Eliane Lages Silva

**Affiliations:** 1 Universidade Federal do Triângulo Mineiro, Instituto de Ciências Biológicas e Naturais, Departamento de Microbiologia, Imunologia e Parasitologia, Uberaba, MG, Brasil.

On July 4, 2020, we said farewell to Egler Chiari, honorable parasitologist, globally renowned Chagas disease researcher, and, affectionately, “Professor” to everyone around him, especially students and staff. At 86 years old, lucid, and still contributing to the study of Chagas disease, he was suddenly taken by COVID-19.

Egler Chiari was born on February 7, 1934, in Belo Horizonte, Minas Gerais, where he lived his entire life. The son of Ervald Chiari and Cecília Chiari, Mr. Chiari married Cléa de Andrade Chiari, with whom he had two children. Later, he enjoyed a stable relationship with Lúcia Maria da Cunha Galvão, his partner until his untimely demise. He started elementary education in a public school-Caetano Azeredo-and completed elementary and high school education at Marconi school; he finished his basic education in 1955. 

**Figure:**
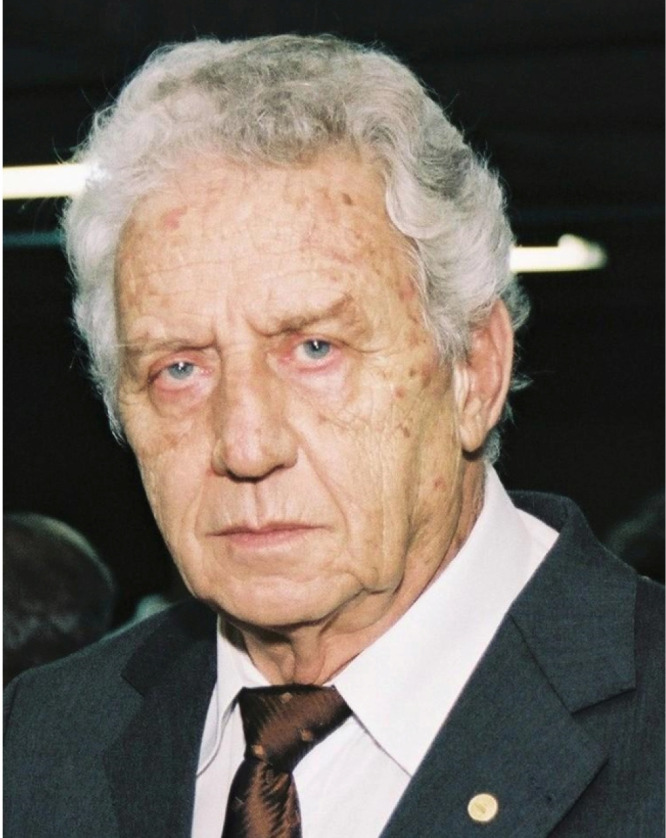

Interestingly, his exposure to scientific research and parasitology preceded his university education. The young Egler showed interest in biology when he passed a public service entry test in 1956 and started working at the currently named René Rachou/FIOCRUZ/ MINAS Institute, Ministry of Health, Belo Horizonte, MG, where he worked for 16 years, initially as a laboratory technician and later as a biology researcher. In this institution, his relationship with Prof. Zigman Brener awakened his scientific spirit and influenced his professional choices. 

He majored in Pharmaceutical Chemistry at the Federal University of Minas Gerais (UFMG) in 1963; during his studies, he worked as a monitor and higher education instructor of Zoology and Parasitology at the UFMG School of Dentistry and Pharmacy. He received his master’s degree (1971) and doctor of Sciences/ Parasitology (1981) from the Postgraduate Course in Parasitology, Department of Parasitology, Institute of Biological Sciences (ICB), UFMG, being supervised by Dr. Zigman Brener and Dr. Erney Felício Plessmann de Camargo, respectively. 

Mr. Chiari started his academic career at UFMG as Assistant Professor of Zoology and Parasitology at the School of Pharmacy (1968) and was assigned to the Parasitology Department of the Institute of Biological Sciences after its formation in 1970. He was Adjunct Professor from 1970 to 1990, became a Full Professor through a public service entry exam (1991), and received the title of Professor Emeritus at UFMG in 2005. He worked as a Parasitology professor in undergraduate courses throughout this period until his retirement in 1992, and continued teaching and researching until 2019 at the UFMG Graduate Program in Parasitology, which he founded, coordinated (1977-1980) and sub-coordinated (1988-1992). He was also a Parasitology professor at the School of Medicine of Itajubá, Itajubá/MG (1971-1972) and at the School of Medicine and Surgery of Uberlândia, Uberlândia/MG (1971-1973). In 2007, he began developing new projects in an endemic area for *Trypanosoma cruzi* in the Potiguar semiarid region, where he set up a research center on Chagas disease. He also contributed financially, academically, and scientifically to the Graduate Programs in Pharmaceutical Sciences and Parasitic Biology of the Federal University of Rio Grande do Norte, training faculty in parasitology.

Chiari, as he was internationally known in the academe, dedicated his life to the study of protozoology directed to the biological, biochemical, and molecular characterization of *Trypanosoma cruzi* and the parasitological diagnosis and experimental chemotherapy of Chagas disease. He was a 1A Researcher and received a PQ/CNPq Research Productivity grant from 1988 to 2016. His restless mind, creative and intellectual capacity, and great determination were embodied by his prolific body of research, which started with the publication of three articles before he started University. Between 1956 and 2019, he published 186 articles in high impact magazines with national and international circulations and seven book chapters. He has been cited 9,302 times (h-index = 53 and i10-index = 144). 

In the 1960s, he published six articles in collaboration with Dr. Zigman Brener, highlighting “Morphological variations in different *Trypanosoma cruzi* samples”. This article published in 1963 in the Journal of the São Paulo Institute of Tropical Medicine is his fourth most cited articles (292 citations). Another important contribution at that time was his study on the viability of the blood culture technique to confirm the chronic phase of Chagas disease. In 1974, he started working as an independent researcher at UFMG, conducting in vitro and in vivo studies on the biology and behavior of *T. cruzi* culture forms. It was also then that he developed one of his most important scientific contributions to the study of human Chagas disease: “A new blood culture technique for the parasitological diagnosis of Chagas disease in the chronic phase” ^1^. Subsequently, this technique was widely used to evaluate studies on the treatment of human and experimental infection to isolate parasite populations and as an important reference in the standardization of molecular PCR and qPCR techniques to diagnose Chagas disease. 

Professor Egler always followed the progression of science, and the following decades were characterized by the consolidation of his master’s and doctor’s degree guidelines, different partnerships, and innovative approaches to his lines of research. The use of cloned *T. cruzi* populations to study parasite variability resulted in his second most cited publication (393 citations), published in 1980 in the Proceedings of the National Academy of Sciences of the United States of America: “Strains and Clones of *Trypanosoma cruzi* can be characterized by Pattern of Restriction Endonuclease Products of Kinetoplast DNA Minicircles^2^”. The advent of molecular biology redirected his line of research. Beginning in 1992, his studies focused mainly on topics such as the identification and evaluation of genetic *T. cruzi* population variability in the blood and tissue of Chagas patients, differentiation of *T. cruzi* and *Trypanosoma rangeli*, diagnosis of human Chagas disease, and evaluation after treatment. Several of his publications in the 1980s and 1990s and his third most cited publication-the 2006 article published in PLoS Pathogens “Ancestral genomes, sex, and the population structure of *Trypanosoma cruzi*”-are pillars of the current classification of *T. cruzi* in six taxonomic groups or Discrete Typing Units (DTUs) conducted by International Consensus in 2009. His most cited article, “A new consensus for *Trypanosoma cruzi* intraspecific nomenclature: second revision meeting recommends TcI to TcVI^3^”, which was published in the Memórias do Instituto Oswaldo Cruz Journal in 2009, has been cited in 975 publications.

Doctor Chiari’s contributions to the academe extended past his own research. He also trained other scientists even after he retired, supervising fourteen master’s theses, thirteen doctoral dissertations, four postdoctoral studies, and countless other scientific studies and undergraduate monographs from 1974 to 2018. He molded several generations of professors and researchers who now work in excellence groups in Graduate Programs in several federal institutions in Brazil (UFMG, UFOP, UFTM, UFRN, UFGO, UEM, FIOCRUZ) and abroad (Colombia). 

Through the course of his academic and scientific career, Professor Egler was honored with several titles, such as Professor Emeritus of the Federal University of Minas Gerais and Full Member of the Brazilian Academy of Sciences. He received numerous medals and plaques in recognition of his exemplary dedication and invaluable scientific contribution to the study of Chagas disease and Brazilian Parasitology. Some of them include the Carlos Chagas Medal, Medal of the Centenary of the Oswaldo Cruz Institute, commemorative plaque of the Centenary of the Discovery of Chagas Disease, and of the 41 years of the Graduate Program in Parasitology ICB/UFMG. He actively participated in scientific committees of several institutions: including the National Research Council (CNPQ), Minas Gerais Research Support Foundation (FAPEMIG), Rio de Janeiro Research Support Foundation (FAPERJ), and Oswaldo Cruz Foundation (FIOCRUZ). He was an ad hoc advisor for different research promotion agencies-such as CNPq, CAPES, FINEP, FAPEMIG, FAPERJ, and FAPESP-and for scientific manuscripts for national and foreign scientific journals-including Memórias do Instituto Oswaldo Cruz, Sociedade Brasileira de Medicina Tropical, Instituto de Medicina Tropical de São Paulo, Acta Tropica, Journal Antimicrobial Chemotherapy, Clinical Infectious Disease, Emerging Infectious Disease, British Journal of Pharmacology, and Journal of Tropical Medicine.

 He was a member of different scientific societies such as the Brazilian Society of Tropical Medicine, Brazilian Society of Parasitology, and Brazilian Society of Protozoology. He was a representative at the Didactic Coordination Board of the Graduate Course in Parasitology, ICB/UFMG, UFMG Graduate Council (1980) and participated in public service entrance exam commissions in different federal institutions (UFMG, USP, UFRN, UFOP, UFTM, FIOCRUZ). In addition to being an organizer of several national scientific events and presented numerous lectures and abstracts in conferences and national and international scientific meetings. 

His passing leaves a great void in the scientific community but not in our lives. He will be remembered for his cheerful, excited, and loud speech; for his restless hands, full of gestures-reflections of his paternal Italian origin-; and for his determination, firmness, justice, and sincerity-a maternal Swiss/German influence-that bothered those who did not truly know him. Ultimately, the dignity of his soul and his concern and affection for his neighbors were privileges for those with whom he shared good wine and his culinary specialty, tenderloin. There’s no way to sad, but we will miss him eternally...
